# Plasminogen Activator Inhibitor 1 Is a Novel Faecal Biomarker for Monitoring Disease Activity and Therapeutic Response in Inflammatory Bowel Diseases

**DOI:** 10.1093/ecco-jcc/jjad160

**Published:** 2023-09-26

**Authors:** Boldizsár Jójárt, Tamás Resál, Diána Kata, Tünde Molnár, Péter Bacsur, Viktória Szabó, Árpád Varga, Kata Judit Szántó, Petra Pallagi, Imre Földesi, Tamás Molnár, József Maléth, Klaudia Farkas

**Affiliations:** Ladon Therapeutics Ltd, Szeged, Hungary; Department of Medicine, University of Szeged, Szeged, Hungary; ELKH-USZ Momentum Epithelial Cell Signaling and Secretion Research Group, University of Szeged, Szeged, Hungary; HCEMM-USZ Molecular Gastroenterology Research Group, University of Szeged, Szeged, Hungary; Department of Medicine, University of Szeged, Szeged, Hungary; Faculty of Medicine, Institute of Laboratory Medicine, University of Szeged, Szeged, Hungary; Department of Medicine, University of Szeged, Szeged, Hungary; ELKH-USZ Momentum Epithelial Cell Signaling and Secretion Research Group, University of Szeged, Szeged, Hungary; HCEMM-USZ Molecular Gastroenterology Research Group, University of Szeged, Szeged, Hungary; Ladon Therapeutics Ltd, Szeged, Hungary; Department of Medicine, University of Szeged, Szeged, Hungary; ELKH-USZ Momentum Epithelial Cell Signaling and Secretion Research Group, University of Szeged, Szeged, Hungary; HCEMM-USZ Molecular Gastroenterology Research Group, University of Szeged, Szeged, Hungary; Department of Medicine, University of Szeged, Szeged, Hungary; ELKH-USZ Momentum Epithelial Cell Signaling and Secretion Research Group, University of Szeged, Szeged, Hungary; HCEMM-USZ Molecular Gastroenterology Research Group, University of Szeged, Szeged, Hungary; Department of Medicine, University of Szeged, Szeged, Hungary; Ladon Therapeutics Ltd, Szeged, Hungary; Department of Medicine, University of Szeged, Szeged, Hungary; ELKH-USZ Momentum Epithelial Cell Signaling and Secretion Research Group, University of Szeged, Szeged, Hungary; HCEMM-USZ Molecular Gastroenterology Research Group, University of Szeged, Szeged, Hungary; Faculty of Medicine, Institute of Laboratory Medicine, University of Szeged, Szeged, Hungary; Department of Medicine, University of Szeged, Szeged, Hungary; Ladon Therapeutics Ltd, Szeged, Hungary; Department of Medicine, University of Szeged, Szeged, Hungary; ELKH-USZ Momentum Epithelial Cell Signaling and Secretion Research Group, University of Szeged, Szeged, Hungary; HCEMM-USZ Molecular Gastroenterology Research Group, University of Szeged, Szeged, Hungary; Department of Medicine, University of Szeged, Szeged, Hungary

**Keywords:** Inflammatory bowel disease, faecal marker, PAI-1

## Abstract

**Background and Aims:**

Crohn’s disease [CD] and ulcerative colitis [UC] require lifelong treatment and patient monitoring. Current biomarkers have several limitations; therefore, there is an unmet need to identify novel biomarkers in inflammatory bowel disease [IBD]. Previously, the role of plasminogen activator inhibitor 1 [PAI-1] was established in the pathogenesis of IBD and suggested as a potential biomarker. Therefore, we aimed to comprehensively analyse the selectivity of PAI-1 in IBD, its correlation with disease activity, and its potential to predict therapeutic response.

**Methods:**

Blood, colon biopsy, organoid cultures [OC], and faecal samples were used from active and inactive IBD patients and control subjects. Serpin E1 gene expressions and PAI-1 protein levels and localisation in serum, biopsy, and faecal samples were evaluated by qRT-PCR, ELISA, and immunostaining, respectively.

**Results:**

The study population comprised 132 IBD patients [56 CD and 76 UC] and 40 non-IBD patients. We demonstrated that the serum, mucosal, and faecal PAI-1 concentrations are elevated in IBD patients, showing clinical and endoscopic activity. In responders [decrease of eMayo ≥3 in UC; or SES-CD  50% in CD], the initial PAI-1 level decreased significantly upon successful therapy. OCs derived from active IBD patients produced higher concentrations of PAI-1 than the controls, suggesting that epithelial cells could be a source of PAI-1. Moreover, faecal PAI-1 selectively increases in active IBD but not in other organic gastrointestinal diseases.

**Conclusions:**

The serum, mucosal, and faecal PAI-1 concentration correlates with disease activity and therapeutic response in IBD, suggesting that PAI-1 could be used as a novel, non-invasive, disease-specific, faecal biomarker in patient follow-up.

## Graphical Abstract

**Graphical Abstract:**
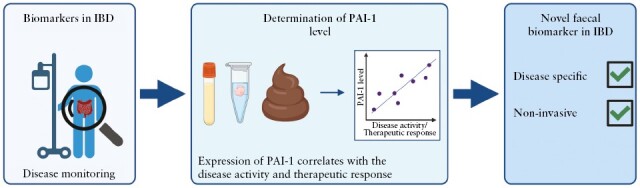

## 1. Introduction

Crohn’s disease [CD] and ulcerative colitis [UC], two forms of inflammatory bowel disease [IBD], are chronic, tissue-destructive conditions developing as a consequence of uncontrolled activation of effector immune cells in the intestinal mucosa. The continuing increase in IBD incidence worldwide is associated with a rapidly increasing global CD and UC burden.^1^ The impact of symptoms and complications on the patients can be profound, leading to impaired quality of life and social and occupational dysfunction.^2,3^ The past decades have brought substantial advances in the pharmacological management of IBD by introducing immunosuppressive agents, biologics, and lately small molecules. However, the sustained efficacy of the currently available anti-inflammatory therapies is still far from optimal. Moreover, an effective treatment may achieve clinical remission, but the subclinical inflammation can persist within the mucosa of the gut, contributing to a risk of ‘symptomatic’ relapse. The tight disease monitoring strategy based on objective inflammation markers and timely therapy escalation leads to improved clinical and endoscopic outcomes, compared with symptom-driven management alone.^4^ In the long term, this approach would decrease the disease burden on patients and the costs of the health care system.^5^ Therefore, an effective monitoring strategy enabling physicians to make difficult decisions to prevent severe disease complications is still an unmet clinical need.

C-reactive protein [CRP] and faecal calprotectin [FC]^6^ are the most widely studied inflammatory biomarkers for disease monitoring. Although CRP is commonly used, it has insufficient sensitivity and specificity for intestinal inflammation.^7^ Recently, faecal biomarkers have emerged as non-invasive, rapid, simple, and low-cost diagnostic tools to detect intestinal inflammation.^8^ Most studies have been performed with FC, which is today the gold standard among faecal markers^9^; however, significant limitations hinder its use in everyday practice.^10^ Therefore, alternative biomarkers must be developed to overcome these shortcomings, precisely monitor the disease course, and predict therapeutic response. In a recent paper, Kaiko *et al.* identified a novel protein—plasminogen activator inhibitor 1 [PAI-1]—showing an important role in controlling key inflammatory modulators during mucosal damage in colitis.^11^ They also highlighted that the mucosal SerpinE1 gene [the gene encoding PAI-1] expression is elevated in active, more severe IBD patients who do not respond to anti-tumour necrosis factor [TNF] therapy. In another study, Wang *et al*. showed that PAI-1 induces neutrophil-mediated chemokine expression by activating the NF-κB pathway in dextran sodium suphate [DSS]-induced colitis model in mice.^12^ These studies emphasised the potential role of PAI-1 in IBD pathogenesis; however, the protein levels of PAI-1 in different biological samples and their correlation with the disease activity or therapeutic response remained unknown.

Therefore, in this study, we aimed to comprehensively analyse the expression profile of PAI-1 in IBD patients’ serum, mucosa, and faeces to investigate its selectivity, correlation with the disease activity, and potential to predict therapeutic response in IBD.

## 2. Patient and methods

### 2.1. Patient population and disease activity measurements

Consecutive patients diagnosed with CD and UC who underwent colonoscopy between 2020 and 2021 at the Department of Medicine, University of Szeged, were enrolled in the study. All IBD patients were eligible for the study except those under 18 years of age, pregnant women, and those who did not consent to take part in the study. The inactive IBD group consisted of patients showing no sign of activity at the index colonoscopy. For patients in the active IBD group, new therapeutic agent[s] such as conventional anti-inflammatory drugs (mesalazine, corticosteroid, budesonide, azathioprine, ciclosporin], anti-cytokine (anti TNF-α [infliximab, adalimumab], anti-IL12/23 [ustekinumab]), α4β7 anti-integrin agent [vedolizumab], or the small molecule JAK inhibitor [tofacitinib] were introduced. Alternatively the current therapy was modified according to the step-up or accelerated step-up strategy, and/or combination therapy was used to increase the therapeutic effectiveness.

Throughout the 12-month follow-up period, patients were strictly controlled at regular visits performed every 2 months. Demographic data, data on disease activity, and therapeutic response were documented at every visit. In CD patients, clinical disease activity was determined using the Crohn’s Disease Activity Index [CDAI] and in patients with UC, clinical disease activity was measured using the partial Mayo score [pMayo].^13,14^ Clinically active disease was defined as CDAI ≥150 in CD and pMayo ≥2 in UC. Colonoscopy was performed at inclusion and Week 52, except for the case of uncertain disease exacerbation requiring unscheduled endoscopy to clarify symptoms. Endoscopically active disease was defined as SES-CD ≥3 in CD and eMayo ≥2 in UC in any examined bowel segment.^15^ Patients were grouped into the following categories based on the clinical and endoscopic activity of their disease, and for simplicity, these groups were used later: inactive, mildly, moderately, and severely active. Regarding the clinical condition of the patients, inactive disease was defined as CDAI˂150 in CD and as pMayo˂2 in UC. Mildly active disease was defined as CDAI between 150 and 219 and pMayo between 2 and 4. CDAI between 220 and 450 and pMayo 5–6 corresponded to moderately active disease, and CDAI ˃450 and pMayo 7–-9 to severe disease.^14,15^ Regarding endoscopic disease activity, SES-CD between 0 and 2 suggested inactive, 3–6 mildly active, 7–15 moderately active, and ≥16 severely active CD.^16^ In UC, eMayo 0–1 expressed inactive, 2 moderately active, and 3 severely active disease.^15^ In the case of active disease, a decrease of the score by 3 or more was taken as a therapeutic response in UC, and a decrease in SES-CD by >50% was considered an endoscopic response in CD.^16^ The control group consisted of non-IBD subjects who underwent colonoscopy.

### 2.2. Sample collection

Biopsies were obtained from the inflamed [from the edge of the ulcer or, in the absence of an ulcer, from the most inflamed region: *n* = 6] and from the non-inflamed part of the colon [*n* = 6] of IBD patients and the healthy sigmoid colon of controls. The biopsies were discharged from the forceps into the transport media. The tissue samples were placed immediately in ice-cold NaHCO_3_ containing Hank’s Balanced Salt Solution [HBSS, Sigma, H9269] and transferred to the laboratory. Serum and faecal specimens were collected within 1–3 days before endoscopy. Serum samples were obtained to determine the routine inflammatory parameters [CRP, total blood count] at every appointment. CRP was considered to be elevated above 5 mg/L. At the same time, additional blood and stool samples were collected for the cytokine profile determination. Blood specimens were centrifuged, and the serum was snap-frozen and stored at -80°C until use. Faecal samples were also frozen until further investigations.

### 2.3. Human colonic organoid culture

Human colonic organoid cultures [OC] were generated using 3–4 colon biopsy samples.^17^ First, colonic crypts were isolated as previously described.^18^ Briefly, biopsy samples were washed three times with HBSS supplemented with an antibiotic and antimycotic mix [Supplementary Table 1]. Then, biopsies were minced into ~1 mm^3^ pieces, transferred to the sterile, 30-ml centrifuge tube [Greiner, 201170], and washed with HBSS 8–10 times. The tissue pieces were placed into 3 ml 10 mM dithiothreitol [DTT, Roche, 11583786001] in completed HBSS to reduce the disulphide bonds, and were incubated in a shaking incubator at 165 rpm at 37°C for 15 min. Next, DTT was removed, and the tissue was washed with HBSS 3 times. The samples were put into 3 ml completed HBSS containing 0.8 mg/ml Collagenase A [Roche, 37170821] and incubated for 50 min at 165 rpm at 37°C in a shaking incubator. At the end of the incubation, the samples were resuspended, and the isolated crypts in the supernatant were checked with a Primovert light microscope [Zeiss]. If necessary, the 15 min digestion step was repeated to maximise the number of isolated crypts. Then, the supernatant was collected into a sterile 1.5 ml tube and centrifuged for 5 min at 2000 rpm at 4°C. The isolated crypts were resuspended in Matrigel [Corning, 356232] diluted with HBSS in a 1:4 ratio, and 10 μl domes were placed into a 24 well plate [Greiner, 662160], two domes per well. After the polymerisation of Matrigel, 1 ml feeding medium [Supplementary Table 2], completed with 10 µM Rho kinase inhibitor [Tocris, 1254], was added per well. The medium was changed every other day. OCs were passaged after 7 days of culture using TrypLE Express Enzyme, completed with 10 µM Rho kinase inhibitor.

### 2.4. Protein isolation from biological samples

For total protein isolation, colonic biopsy samples were minced into small pieces with a razor blade in a Petri dish and were transferred into 1 ml RIPA lysis buffer [Millipore, 20-188]. The 10 × RIPA lysis buffer was diluted in AccuGene water [Lonza, 51200] and completed with EASYpack Protease Inhibitor Cocktail [Roche, 05892970001]. The biopsy samples were homogenised with a Branson Sonifier SFX150 on ice [four cycles, 10 s sonication, and 10 s break per cycle]. After that, the samples were centrifuged at 3500 rpm for 10 min at 4°C. The supernatant was transferred into a new 1.5 ml tube, snap-frozen, and stored at -80°C until use. For total protein isolation from stool samples, 100 mg stool was vortexed in 300 µl RIPA lysis buffer with protease inhibitor for 5 min. After that, the samples were centrifuged at 4000 *g* for 10 min at 4°C. The supernatant was transferred to a new, sterile tube, and the centrifuge step was repeated at 12000 *g* for 10 min at 4°C. The supernatant was transferred into a new 1.5 ml tube, snap-frozen, and stored at -80°C until use.

### 2.5. Determination of the cytokine expression in serum, colonic biopsy, and faecal samples

To define the cytokine expression pattern of the serum and the colonic mucosa, the Proteome Profiler Human Cytokine Array Kit [R&D Systems, ARY005B] was used according to the manufacturer’s protocol. Briefly, 200 µl sera were used without dilution in all serum cytokine determination measurements; for the mucosal cytokine expression investigation, 500 µg total protein was applied at all membranes after total protein isolation. The total protein concentrations of biopsy samples were determined by Bradford assay. Blots were imaged with a ChemiDoc MP [BioRad].

### 2.6. Determination of PAI-1 in serum, colonic biopsy, organoids, and faecal samples

To determine the plasminogen activator inhibitor 1 [PAI-1] concentration in the serum, mucosa, and faecal samples, a commercially available enzyme-linked immunosorbent assay [ELISA] kit [Abcam, ab269373] was used according to the manufacturer’s protocol. Multiskan FC [Thermo Scientific] plate reader was used for the ELISA and Protein Bradford measurements.

#### 2.6.1. Serum

Blood specimens were collected and stored as described above. For serum ELISA measurements, samples were diluted to 20–100 x with the Sample Diluent NS buffer of the ELISA kit.

#### 2.6.2. Tissue and organoids

For the tissue and organoid samples, the total protein concentrations were defined by the Protein Bradford method and protein levels were normalised to the total protein concentration.

#### 2.6.3. Faeces

For the faecal samples, the total protein concentrations were defined by the Protein Bradford method, and protein levels were normalised to the total protein concentration. For the measurement of PAI-1 in tissue and faecal samples, the ab269373 ELISA kit was validated. Recombinant human PAI-1 protein from the ab269373 ELISA kit was used for the recovery measurements, and the following quantities of PAI-1 were added to the samples: 0, 15.6, 31.25, 62.5, 125, 250, 500, and 1000 pg. Three biological and three technical measurements were done at all sample types according to the manufacturer’s protocol. The results of the validation are summarised in Supplementary Figure 1**.**

### 2.7. Gene expression analyses of colonic biopsy samples and organoid cultures

Gene expression analyses were performed as previously described.^17^ Briefly, total RNA was isolated from homogenised biopsies with NucleoSpin RNA Plus Kit [Macherey-Nagel, 740984], whereas for homogenised organoids NucleoZOL [Macherey-Nagel, 740404.200] was used according to the instructions of the manufacturer. iScript cDNA Synthesis Kit [Bio-Rad, 1708891] was used for the reverse transcription of mRNA samples. The cDNA concentration was 50 ng/μl at the tissue and 25 ng/μl at the OC samples. The primers were designed in the NCBI Primer-BLAST [Supplementary Table 3]. Except for BACT, all primers were designed to the exon-exon junction, and all polymerare chain reaction [PCR] products were less than 200 bp. Amplicons were detected by a Lightcycler 96 [Roche, 05815916001] using SYBR Green [Bio-Rad, 1725274]. GAPDH and BACT were used as housekeeping genes for normalisation, and the Livak Method was applied to the evaluation. The gene expression of non-IBD control patients was applied to define the relative gene expression fold changes of IBD patients.

### 2.8. Immunofluorescence staining

Immunostainings were performed as previously described.^19^ Briefly, colon biopsies were fixed in 4% PFA-PBS for 2 h and then incubated overnight in 30% sucrose for cryoprotection. Fixed biopsy samples and organoids were embedded into Cryomatrix [Thermo Scientific, 6769006], frozen at -20°C, and 7-μm thick sections were cut. Organoids were fixed after sectioning in 4% PFA-DPBS for 20 min at room temperature. Biopsies were permeabilised with 0.02% Triton-X100-TBS for 10 min, followed by 10 min of washing in TBS three times. For organoids, the permeabilisation was performed using citrate-Tween 20 solution (0.001 M sodium citrate buffer [Sigma, C8532], pH 6.0, and 0.05% Tween 20 [Sigma, P1379-100]) and boiled for 30 min. Unspecific antibody binding was blocked for 2 h at 37°C with 10% BSA-TBS containing 1% goat serum. Mouse monoclonal anti PAI-1 primary antibody [Invitrogen, MA-33H1F7] was diluted 1:200 in 10% BSA-TBS, and sections were incubated overnight at 4°C. The primary antibody was washed three times as described, and then an Alexa Fluor Plus 488 conjugated goat anti-mouse secondary antibody [Invitrogen, A48286] was applied in 1:2000 dilution for 2 h at room temperature. Samples were washed three times, and then nuclei were labelled with DAPI [Sigma, MBD0015-5ML] in 1:500 dilution for 30 min. For isotype control, mouse polyclonal IgG [ab37355] under the same conditions as the primary antibody was used. Sections were placed into Fluoromount [Sigma, F4680-25ML], and images were obtained by Zeiss LSM 880 confocal microscope with a 40 x oil immersion objective.

### 2.9. PAI-1 stability in faecal samples

The stability of the PAI-1 was defined in stool samples; 100 mg faeces was diluted into 1.5 ml sterile tubes and incubated at room temperature and 4°C for up to 7 days. Total protein isolation was performed every day. The isolation protocol and the PAI-1 level determination were the same as described above.

### 2.10. Data and statistical analysis

All software used for data analysis is summarised in Supplementary Table 4. Statistical analysis was performed with GraphPad Prism software [GraphPad, 9.5.0]. For all data with non-normal distribution, non-parametric tests were employed. Between two groups, unpaired Mann–Whitney or paired Wilcoxon tests were performed. The Kruskal–Wallis test with Dunn’s multiple comparisons test was used for three or more groups. The significance was accepted at *p* <0.05. Data are imaged by box plot and presented with the median, interquartile range [IQR], and minimum and maximum points. For the correlation analysis, non-parametric Spearman’s correlation was used. MedCalc statistical software [MedCalc Software, 20.211] was used for the receiver operating characteristic [ROC] analysis. The area under the curve [AUC] value, sensitivity, and specificity were defined, and the cut-off value was determined according to the maximisation criterion of the Youden index.

### 2.11. Ethical considerations

Ethical approval for the study was obtained from the Regional and Institutional Human Medical Biological Research Ethics Committee, University of Szeged [247/2018-SZTE; 4412]. The research was carried out according to the Code of Ethics of the World Medical Association [Declaration of Helsinki], and written informed consent was obtained from the enrolled patients.

## 3. Results

### 3.1. Patient population

The study population consisted of 132 IBD patients [56 CD and 76 UC] and 40 non-IBD patients [10 without abnormalities, seven with diverticulosis, 14 with colon polyps, and nine with colorectal cancer]. The demographic and clinical characteristics of the IBD patients are presented in Table 1 and Table 2. The mean age was 39 years for both CD [22–72] and UC [18–72]. Patients in clinical remission accounted for 37.5% of CD and 28.9% of UC, and endoscopic remission was confirmed in 30.4% of CD and 25% of UC patients. New therapy was introduced in 21.4% of CD and 21.1% of UC patients. Switch and dose escalation of biological therapy was carried out in 16.1% and 14.3% of patients with CD and 17.1% and 9.2% of patients with UC.

**Table 1.** Demographic and clinical characteristics of the study population.

	Crohn's disease	Ulcerative colitis
Number, **n**	56	76
Female, *n* [%]	27 [48%]	36 [47.4%]
Age [mean, years]	39	39
Disease duration [mean, years]	11	9.2
**CD location, *n* [%]**		
Ileal [L1]	9 [16.1%]	-
Colonic [L2]	24 [42.8%]	-
Ileocolonic [L3]	23 [41.1%]	-
**UC extent, *n* [%]**		
Proctitis [E1]	-	4 [5.3%]
Left-sided [E2]	-	36 [47.4%]
Pancolitis [E3]	-	36 [47.4%]
**Clinical disease activity at inclusion, *n* [%]**		
Inactive	21 [37.5%]	22 [28.9%]
Mild	14 [25%]	12 [15.8%]
Moderate	13 [23.2%]	27 [35.5%]
Severe	8 [14.3%]	15 [19.7%]
**Endoscopic activity at incusion, *n* [%]**		
Inactive	17 [30.4%]	19 [25%]
Mild	6 [10.7%]	9 [11.8%]
Moderate	13 [23.2%]	31 [40.8%]
Severe	20 [35.7%]	17 [22.4%]
**Failure to previous treatment, n [%]**		
Immunomodulator	14 [25%]	8 [10.5%]
Anti-TNF	22 [39.3%]	35 [46%]

TNF, tumour necrosis factor.

**Table 2.** Summary table of the medications applied in the study population.

	Crohn's disease	Ulcerative colitis
**Medication at inclusion, n [%]**		
Nothing	10 [17.9%]	6 [7.9%]
5-aminosalicylic acid	1 [1.79%]	27 [35.5%]
Corticosteroids	3 [5.36%]	13 [17.1%]
Azathioprine/6-mercaptopurine	10 [17.9%]	16 [21.1%]
Cyclosporine	1 [1.79%]	4 [5.3%]
Anti-TNF	16 [28.6%]	18 [23.7%]
Vedolizumab	13 [23.2%]	24 [31.6%]
Ustekinumab	10 [17.9%]	1 [1.3%]
Tofacitinib	0	5 [6.6%]
**Medication after colonoscopy, *n* [%]**		
5-aminosalicylic acid	1 [1.79%]	24 [31.6%]
Corticosteroids	10 [17.9%]	10 [13.2%]
Azathioprine/6-mercaptopurine	4 [7.14%]	14 [18.4%]
Cyclosporine	0	2 [2.6%]
Anti-TNF	18 [32.1%]	20 [26.3%]
Vedolizumab	12 [21.4%]	21 [27.6%]
Ustekinumab	17 [30.4%]	1 [1.3%]
Tofacitinib	0	15 [19.7%]
**Treatment change after colonoscopy, *n* [%]**		
New introduction of IM and/or biologic therapy	12 [21.4%]	16 [21.1%]
Continuation	25 [44.6%]	37 [48.7%]
Switch	9 [16.1%]	13 [17.1%]
Dose escalation	8 [14.3%]	7 [9.21%]

TNF, tumour necrosis factor; IM, immunomodulation.

### 3.2. Serum concentration of PAI-1 is elevated in IBD patients and decreases in response to effective therapy

**Figure 1.**
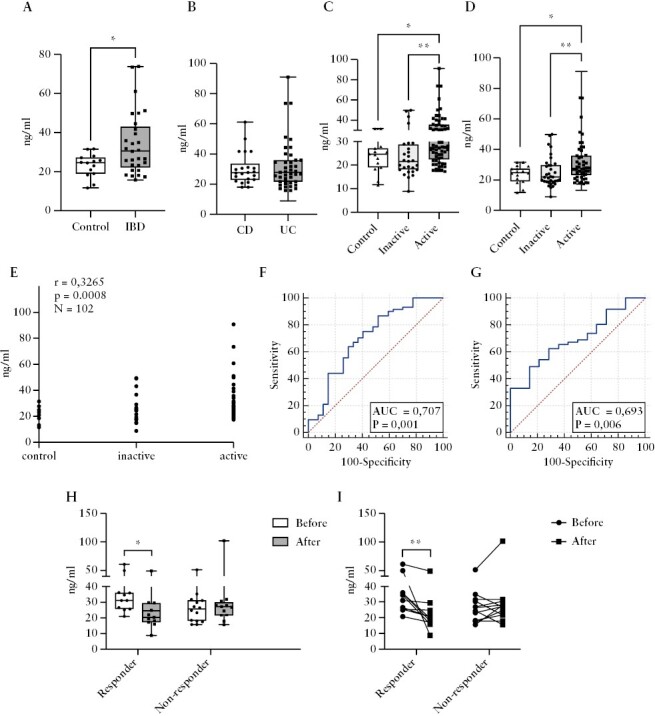
Determination of the PAI-1 level in serum. A. The serum PAI-1 concentration was significantly higher in the patients with IBD [*N *= 29] compared with control [*N* = 14] subjects [*p* = 0.0409]. B. No significant difference was detected in the UC [*N *= 41] and CD [*N* = 22] patients [*p* = 0.9915]. C–D. Significantly increased concentration of PAI-1 was measured in the endoscopically [C] and clinically [D] active IBD [endoscopically: *N* = 61, clinically: *N* = 52] patients compared with the inactive patients [endoscopically: *N* = 27, clinically: *N* = 34] and with controls [N = 14] [endoscopically: inactive vs active *p* = 0.0018, control vs active *p* = 0.0239; clinically: inactive vs active *p* = 0.0069, control vs active *p* = 0.0273]. E. Correlation analysis between disease activity and the serum PAI-1 concentration. F–G. ROC analysis between the inactive [*N *= 27] and active [*N* = 61] IBD [F] and control [*N* = 14] vs active [*N* = 61] IBD patients [G]. H–I. The serum PAI-1 concentration significantly decreased after the induction of therapy in the responders [*N* = 10] [*p* = 0.047 and *p* = 0.002]. *p <0.05; **p <0.01. IBD-Inflammatory Bowel Disease, CD-Crohn's disease, UC-Ulcerative colitis, AUC-Area under curve.

In the study by Kaiko et *al*., PAI-1 has been highlighted as a potential link between the epithelium and inflammation in the pathogenesis of IBD.^11^ The authors also demonstrated the discriminatory power of Serpin E1 [the gene encoding PAI-1] gene expression to distinguish between inflamed IBD biopsies versus non-inflamed/non-IBD biopsies. This observation was also confirmed on the protein expression level in a preliminary multiplex cytokine array, which confirmed the increased expression of PAI-1 in the serum and colon mucosa of IBD patients [Supplementary Figure 2]. Next, we demonstrated that the level of PAI-1 is significantly elevated in the serum samples of IBD patients versus the control subjects [24.62 ng/mL vs 30.48 ng/mL, *p* = 0.0409] [Figure 1A]. PAI-1 levels did not differentiate between UC and CD [27.77 vs 27.44 ng/mL, *p* = 0.99; Figure 1B]. On the other hand, PAI-1 levels were significantly higher in IBD patients with endoscopic [Figure 1C] or clinical activity [Figure 1D] compared with inactive and control patients [endoscopically active vs inactive: 27.77 vs 21.67 ng/mL, *p* = 0.0018, endoscopically active vs control: 27.77 vs 24.62 ng/mL, *p* = 0.0239; clinically active vs inactive: 27.44 vs 21.7 ng/mL, *p* = 0.0069, clinically active vs control: 27.44 vs 24.62 ng/mL *p* = 0.0273]. Of note, CRP was significantly increased in endoscopically or clinically active patients, but in UC patients it was significantly lower than in CD patients [Supplementary Figure 3A–C.]. The increased serum PAI-1 level correlated with the endoscopic activity [r = 0.3265, *p* = 0.0008; Figure 1E]. Furthermore, ROC curves demonstrated that serum PAI-1 showed a moderate power to distinguish between active and inactive IBD patients (area under the curve [AUC] = 0.71; specificity: 48%; sensitivity: 87%; cut-off: 19.99 ng/mL; Figure 1F) and between controls and active IBD patients [AUC = 0.69; specificity: 48%; sensitivity: 87%; cut-off: 27.96 ng/mL; Figure 1G]. Next, we compared the change in PAI-1 levels in patients responding to the anti-inflammatory therapy with non-responders. The PAI-1 level decreased significantly after the induction of therapy in responders, and this was not the case in non-responders [responders: 30.96 ng/mL vs 21.14 ng/mL, *p* <0.0147; non-responders: 26.49 ng/mL vs 26.87 ng/mL, *p* = 0.93] [Figure 1H, I]. Additionally after the treatment, the serum PAI-1 concentration was significantly lower in responders than in non-responders [Supplementary Figure 3D]. However, CRP did not show a significant difference between the responder and non-responder patients [Supplementary Figure 3D]. Furthermore, ROC curves demonstrated that CRP showed a moderate power to distinguish between active and inactive IBD patients [AUC = 0.74; specificity: 96%; sensitivity: 50%; cut-off: 9.3 mg/mL; Supplementary Figure 3E] and between CD and UC patients [AUC = 0.82; specificity: 78%; sensitivity: 74%; cut-off: 15.3 mg/mL; Supplementary Figure 3F].

### 3.3. Serpin E1 gene expression in the colonic biopsies was significantly elevated in IBD patients and decreased in responders

**Figure 2.**
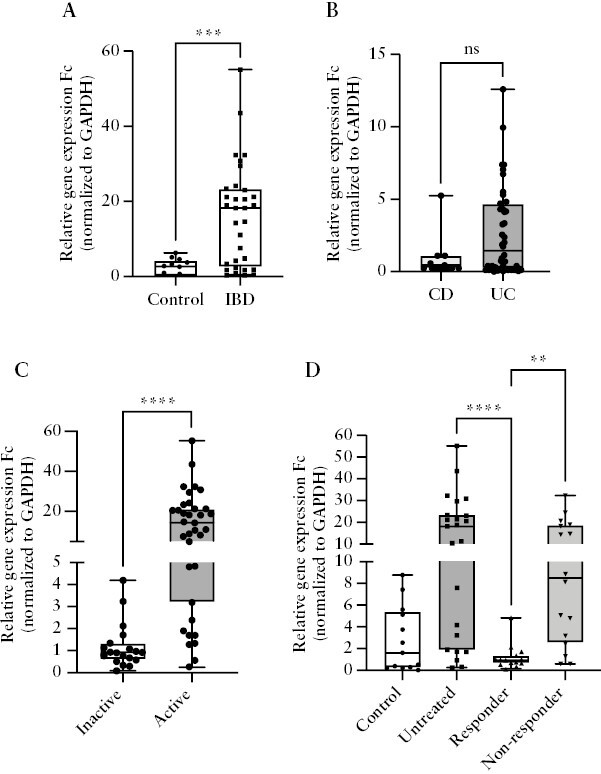
Gene expression pattern of Serpin E1 in mucosa. A. Relative gene expression fold change [Fc] in the IBD patients [*N* = 32] compared with controls [*N* = 13] of the Serpin E1 gene. B. Comparison of the gene expression patterns of Serpin E1 in CD [*N* = 12] and UC patients [*N* = 46]. C. Comparison of the gene expression patterns of Serpin E1 in inactive [*N* = 20] and endoscopically active IBD patients [*N* = 35]. D. Comparison of the gene expression patterns of Serpin E1 in controls [*N* = 13], untreated IBD patients [*N *= 23], responders [*N* = 16], and non-responders [*N* = 16]. *p <0.05; **p <0.01; ***p <0.001. IBD-Inflammatory Bowel Disease, CD-Crohn's disease, UC-Ulcerative colitis, GAPDH-Glyceraldehyde 3-phosphate dehydrogenase.

The primary sources of the secreted PAI-1 in the blood circulation are the endothelial cells, but other cell types, including epithelial and immune cells or adipocytes, also express this protein, which may be more significant in the local inflammation in IBD. Therefore we determined the relative gene expression fold changes [Fc] of Serpin E1 [the gene encoding PAI-1] in colonic biopsy samples from non-IBD control subjects, therapy-naive, active IBD patients, and responders and non-responders. Compared with the control samples, mucosal Serpin E1 expression was significantly higher in therapy-naive, active IBD patients [2.585 vs 18.17, *p* = 0.006; Figure 2A] but showed no significant difference between CD and UC patients [Figure 2B]. We also determined the relative expression of four well-known inflammatory genes, TNF-α, IL-1β, IL-6, and TGF-β, from the same samples. In these experiments, IL-1β and TGF-β showed elevated expression in the untreated IBD biopsies, whereas TNF-α and IL-6 were not significantly elevated [Supplementary Figure 4A, B]. In addition, Serpin E1 gene expression was significantly lower in endoscopically inactive patients compared with active subjects [0.96 vs 14.32, *p* <0.0001, Figure 2C] and in responders compared with the untreated patients [0.9326 vs 18.17, *p* <0.0001], or with non-responders [0.9326 vs 8.487, *p* = 0.002 Figure 2D]. Among the four other genes, only TGF-β and IL-1β were upregulated in the endoscopically active IBD patients and decreased in responder subjects [Supplementary Figure 4C and D].

### 3.4. Colonic mucosal expression of PAI-1 is higher in IBD and decreases significantly in response to the therapy

**Figure 3.**
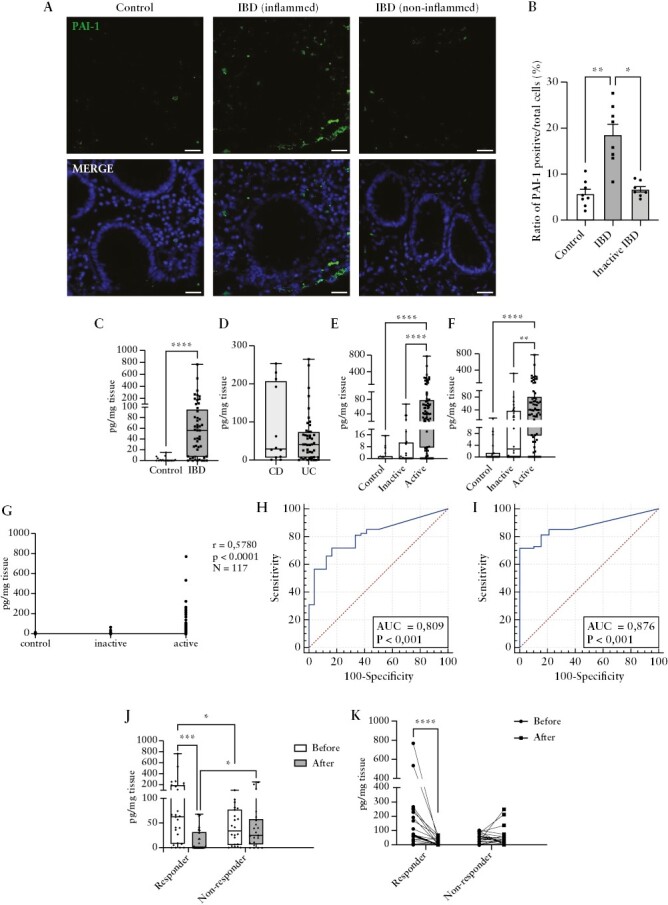
The concentration of PAI-1 in the colonic mucosa. A. Localisation of PAI-1 in the colonic mucosa [blue: nucleus, green: PAI-1, scale bar: 25 µm]. B. Increased ratio of PAI-1 positive cells was detected in the mucosa of inflamed samples [*N* = 8] compared with non-inflamed tissues [*N* = 8, *p* = 0.0126] and controls [*N* = 8, *p* = 0.014]. C. The mucosal PAI-1 concentration was significantly higher in the patients with IBD [*N* = 49] compared with control [*N* = 19] subjects [*p* <0.0001]. D. No significant difference was detected between the CD [*N* = 12] and UC [*N* = 45] patients [*p* = 0.7903]. E–F. Significantly increased concentration of PAI-1 was measured in the endoscopically [E] and clinically [F] active [endoscopically active: *N* = 74, clinically active: *N *= 62] IBD patients compared with the inactive patients [endoscopically inactive: *N* = 24, clinically inactive: *N* = 36] and controls [*N* = 19] [endoscopically inactive vs active *p* <0.0001, control vs endoscopically active p <0.0001; clinically inactive vs active *p* = 0.0015, control vs clinically active p <0.0001]. G. Correlation analysis between the mucosal PAI-1 level and endoscopic disease activity. H–I. ROC analysis of the PAI-1 levels [H] between the inactive [*N* = 24] and active IBD patients [*N* = 74] and [I] between the control [*N* = 19] and active IBD [*N* = 74] subjects. J–K. After the effective therapy, the mucosal PAI-1 concentration decreased in responders [*N* = 26]. *p <0.05; **p <0.01; ***p <0.001; ****p <0.0001. IBD-Inflammatory Bowel Disease, CD-Crohn's disease, UC-Ulcerative colitis, PAI-1-Plasminogen activator inhibitor 1

Immunofluorescence staining was performed in biopsy samples to determine the protein expression and localisation of PAI-1 in the colonic mucosa [Figure 3A]. In accordance with the elevated gene expression, the number of PAI-1 positive cells was significantly higher in biopsy samples obtained from the inflamed part of the colon [IBD vs control: 18.52% vs 5.718%, *p* = 0.0014; IBD vs inactive IBD: 18.52% vs 6.651%, *p* = 0.0126], whereas the non-inflamed part showed no difference when compared with control samples [5.716% vs 6.641%, *p* >0.9999; Figure 3B]. Notably, the majority of the PAI-1 positive cells were observed primarily in the epithelial cell layer. Next, we isolated the total protein from the biopsy samples and determined the PAI-1 concentration in the colonic tissue, which was significantly higher in the IBD samples [0.00 vs 55.96 pg/mg, *p* = 0.001; Figure 3C]. However, no significant difference was detected between CD and UC patients [29.24 vs 40.55 pg/mg, *p* = 0.7903; Figure 3D]. Moreover, PAI-1 concentration was significantly higher in patients with endoscopic [active vs inactive: 40.83 vs 0.23 pg/mg, *p* <0.0001; active vs control: 40.83 vs 0.00, *p* <0.0001; Figure 3E] or clinical activity [active vs inactive: 40.55 vs 2.770 pg/mg, *p* = 0.0015; active vs control: 40.55 vs 0.00 pg/mg, *p* <0.0001; Figure 3F] compared with the controls or inactive patients. Of note, the mucosal PAI-1 concentration was not significantly different in inactive patients and controls [not shown]. The increased mucosal PAI-1 level showed a correlation for the endoscopic activity [r = 0.578, *p* <0.0001; Figure 3G]. ROC curves demonstrated that mucosal PAI-1 showed a relatively high power to distinguish between active and inactive IBD patients [AUC = 0.81; specificity: 83%; sensitivity: 72%; cut-off: 18.16 pg/mg; Figure 3H] and between controls and active IBD patients [AUC = 0.88; specificity: 100%; sensitivity: 72%; cut-off: 15.49 pg/mg; Figure 3I]. Finally, when patients were categorised into responder and non-responder groups based on their response to the selected anti-inflammatory therapy, we found that the initial PAI-1 level was significantly higher in the responder patients compared with the non-responders [62.77 vs 34.15 pg/mg, *p* = 0.03; Figure 3J]. Notably, mucosal PAI-1 concentration decreased significantly in responders, and this is not the case in non-responders [responders: 62.77 vs 0.23 pg/mg, *p* = 0.0001; non-responders: 34.15 vs 24.58 pg/mg, *p* = 0.58; Figure 3K]. These results suggest that the tissue concentration of PAI-1 correlates with the disease activity and decreases in response to therapy.

### 3.5. PAI-1 expression is higher in IBD patient-derived colonic organoids compared with controls

**Figure 4.**
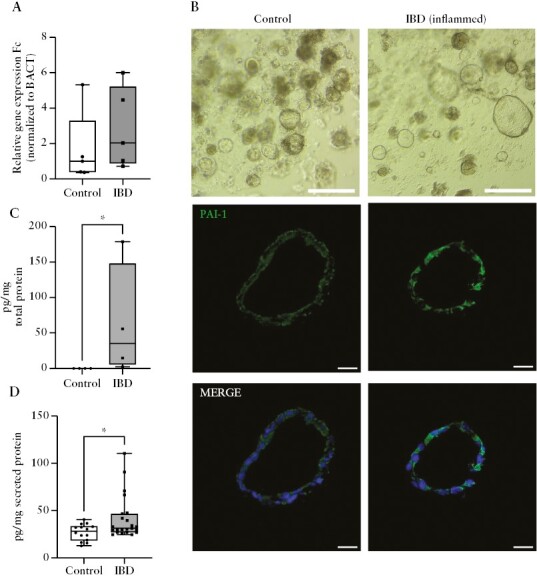
Gene and protein expression of Serpin E1 in human colon organoids. A. Relative gene expression fold change [Fc] of Serpin E1 is not significantly altered in organoid cultures [OCs] generated from active IBD patients [*N* = 4] and controls [*N *= 4] [*p* = 0.3094]. B. Transmission and confocal microscope images of control and IBD OCs. Localisation of the PAI-1 in control and IBD OCs [blue: nucleus, green: PAI-1, scale bar: 25 µm]. C–D. Increased isolated [IBD: *N* = 4, control: *N* = 4, *p* = 0.0286] [C] and secreted [IBD: *N* = 23, control: N = 14, *p* = 0.0423]. [D] PAI-1 levels in the IBD OCs compared with the controls. *p <0.05. IBD-Inflammatory Bowel Disease, BACT-Beta-actin.

To further investigate whether epithelial cells could be the primary sources of PAI-1 in the colonic tissue, we established colonic organoid cultures [OC] from biopsy samples obtained from control subjects and IBD patients with active disease. Adult stem cell-based organoids consist of epithelial cells that display apical to basal polarity and maintain features of the original tissue in 3D cell cultures.^20^ In our experiments, OCs were used for experiments between the first and second passage to avoid changes in the inflammatory phenotype. The analysis of Serpin E1 revealed that the relative gene expression Fc was higher in the inflamed IBD organoids; however, the difference was not significant [2.034 vs 1.003 ng/g, *p* = 0.3095; Figure 4A]. On the other hand, immunostaining revealed that the expression of PAI-1 was higher in IBD organoids [Figure 4B]. Moreover, the PAI-1 concentration in the organoids [35.29 vs 0.00 ng/g, *p* = 0.0286; Figure 4C] and in the medium [31.28 vs 28.50 ng/g, *p* = 0.0423; Figure 4D] were significantly higher in the IBD organoids compared with the control.

### 3.6. PAI-1 as a potential faecal biomarker in IBD

**Figure 5.**
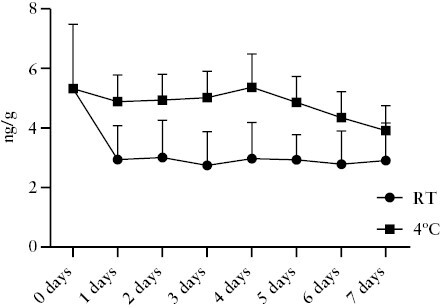
Stability of the faecal PAI-1 at room temperature and 4°C. RT-Room temperature.

**Figure 6.**
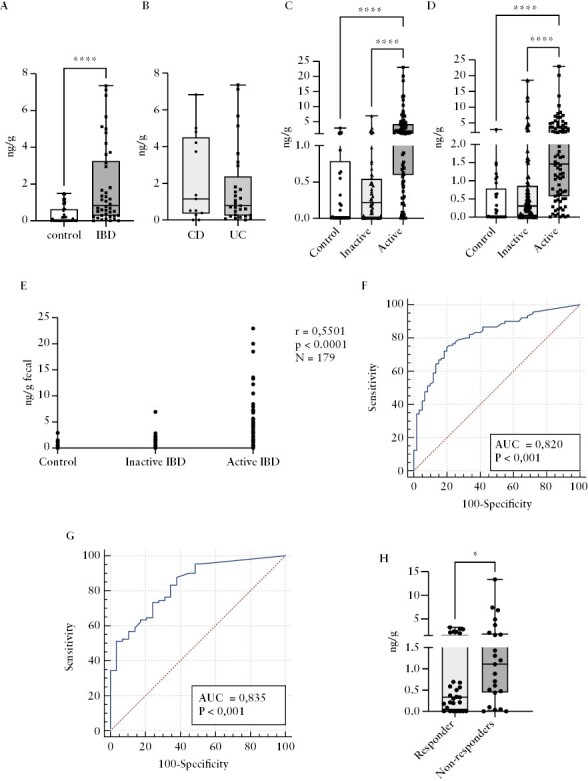
PAI-1 concentration in faecal samples. A. Faecal PAI-1 concentration was elevated in IBD patients [*N* = 42] compared with controls [N = 39] [*p* <0.0001]. B. No significant difference in the faecal PAI-1 levels was detected among the CD [*N* = 13] and UC [*N* = 29] patients [*p* = 0.7319]. C–D. Faecal PAI-1 levels were significantly higher in endoscopically [C] and clinically [D] active IBD patients [endoscopically: *N* = 90, clinically: *N* = 78] compared with the inactive patients [endoscopically: *N* = 60, clinically: *N *= 72] and controls [*N* = 29] [endoscopically: inactive vs active *p* <0.0001, control vs active *p* <0.0001; clinically: inactive vs active *p* <0.0001, control vs active *p* <0.0001]. E. Faecal PAI-1 concentration displayed a positive correlation with the endoscopic disease activity. F–G. ROC analysis of the faecal PAI-1 levels between the inactive [*N* = 60] and active [N = 90] IBD patients [F] and between the control [*N* = 29] and active [*N* = 90] IBD subjects [G]. H. Faecal PAI-1 concentration was elevated in non-responder patients [*N* = 23] compared with responders [*N *= 33] [*p* <0.0001]. IBD-Inflammatory Bowel Disease, CD-Crohn's disease, UC-Ulcerative colitis, AUC-Area under curve.

The widely accepted tight disease monitoring strategy in IBD requires rapid, cheap, and easily accessible biomarkers, allowing patient self-testing. Faecal disease markers are suitable for these goals. Therefore, in the next step we analysed the faecal concentration of PAI-1 in IBD patients. First, we assessed the stability of PAI-1 in the faeces at room temperature and 4°C up to 7 days, as this is a crucial parameter for a faecal biomarker. Our results suggest that PAI-1 remains stable for 7 days at 4°C in faecal samples collected from IBD patients [Figure 5]. In addition, we showed that PAI-1 concentration decreased in the first 24 h at room temperature but remained stable for 7 days. Notably, in these experiments, we did not use any stabiliser or enzyme inhibitor agent, which presumably could further improve the stability of PAI-1. In the next step, we compared the concentration of PAI-1 in faecal samples collected from controls and IBD patients. Our results demonstrated that the level of PAI-1 was significantly higher in the faecal samples of IBD patients in general [0.00 vs 0.84 ng/g, *p* <0.0001; Figure 6A]. Similar to the serum and tissue PAI-1 levels, we could not find any significant difference between UC and CD patients [0.77 vs 1.15 ng/g, *p* = 0.73; Figure 6B]. Importantly, the faecal PAI-1 concentration correlated with the endoscopic and clinical disease activity, and it was significantly higher in patients with endoscopically or clinically active disease compared with inactive patients and controls [endoscopically active IBD vs inactive IBD: 1.535 vs 0.215 ng/g, *p* <0,0001; endoscopically active IBD vs control: 1.535 vs 0.02 ng/g, *p* <0.0001; clinically active IBD vs inactive IBD: 1.45 vs 0.01 ng/g, *p* <0,0001; clinically active IBD vs control: 1.45 vs 0.02 ng/g, *p* <0.0001; Figure 6C, D]. The increased faecal PAI-1 level correlated with the endoscopic activity [r = 0.5501, *p* <0.0001; Figure 6E], whereas ROC curves demonstrated that faecal PAI-1 showed a relatively high power to distinguish between active and inactive IBD patients [AUC = 0.82; specificity: 80%; sensitivity: 74%; cut-off: 0.6 ng/g; Figure 6F] and between controls and active IBD patients [AUC = 0.83; specificity: 62%; sensitivity: 88%; cut-off: 0.2 ng/g; Figure 6G]. In the same patient cohort, CRP was significantly increased in the clinically or endoscopically active patients as well. However, no correlation was detected between CRP and faecal PAI-1 concentration [Supplementary Figure 5A–C]. Finally, the faecal PAI-1 concentration was significantly lower in responders vs non-responders after the treatment [0.33 vs 1.1 pg/g, *p* = 0.047; Figure 6H]. Importantly, CRP did not show a significant difference between the responder and non-responder patients [Supplementary Figure 5D]. These results suggest that faecal PAI-1 could be used as a novel biomarker in the clinical follow-up of IBD patients.

### 3.7. PAI-1 as a potential faecal marker for differential diagnosis of colorectal diseases

**Figure 7.**
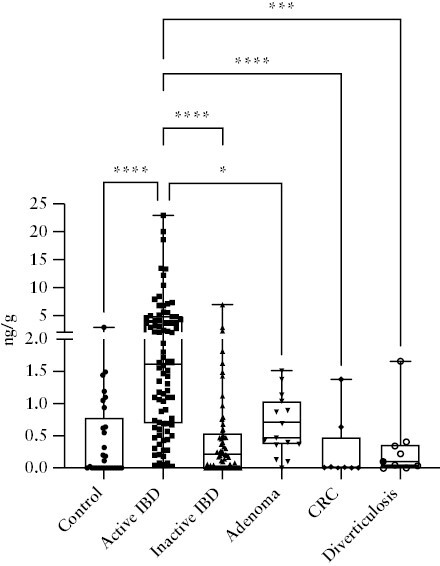
Faecal PAI-1 concentration in organic gastrointestinal diseases. Increased PAI-1 levels were detected in faecal samples of active IBD patients [*N* = 86] compared with patients with organic gastrointestinal diseases [control: *N* = 29, *p* <0.0001; inactive IBD: *N* = 60, *p* <0.0001; adenoma: *N* = 15, *p* = 0.0346; colorectal cancer: *N* = 8, *p* <0.0001; diverticulosis: *N* = 9, *p* = 0.0005]. IBD-Inflammatory Bowel Disease, CRC-Colorectal cancer.

The controversial selectivity is one of the most significant limitations of the current gold standard FC, which could also be elevated in other organic gastrointestinal diseases.^21^ To assess the selectivity of faecal PAI-1, we collected samples from negative controls, IBD patients, and patients who had organic lesions detectable at colonoscopy except for IBD, and allocated them to six groups: negative, active IBD, inactive IBD, adenoma, colorectal cancer, and diverticulosis [without inflammation]. We found that faecal PAI-1 was elevated only in active IBD. In contrast, in other gastrointestinal [GI] diseases such as adenoma, colorectal cancer, or diverticulosis, it did not show a significant elevation compared with the control patients [Figure 7]. To gain information about the dependency on disease localisation, we compared the faecal concentration of PAI-1 in patients with ileal, colonic, or ileocolonic localisations. In this cohort, the faecal PAI-1 concentration was significantly higher in patients with colonic and ileocolonic localisation. Although patients with ileal localisation displayed elevated faecal PAI-1 concentration, the difference was not significant compared with the controls. Notably, there were no significant differences among the faecal PAI-1 concentrations in the ileal, colonic, or combined localisations [Supplementary Figure 6]. Due to the low numbers, these observations require further validation.

## 4. Discussion

PAI-1 emerged recently as a critical link between the epithelium and inflammation, which showed elevated mucosal gene expression in patients with IBD who did not respond to anti-TNF biologic therapy.^11^ The present study demonstrated that the serum, mucosal, and faecal PAI-1 concentration is selectively elevated in IBD patients showing clinical and endoscopic activity but not in other organic gastrointestinal diseases, and decreased significantly upon successful therapy in responders.

PAI-1, encoded by the gene Serpin E1, is a member of the serine protease inhibitor [serpin] family, and its primary function is to inhibit the tissue- and urokinase-type plasminogen activator, which has a major role in the fibrinolysis and the homeostasis of the extracellular matrix.^22^ The expression of PAI-1 is regulated by multiple growth factors, hormones, and inflammatory cytokines [eg, TNF-α, IL-1β, IL-6, and TGF-β],^23–27^ and it can be secreted by different cell types [eg, endothelial, epithelial, and immune cells] and act as a pleiotropic cytokine.^28^ Clinical studies have established that patients with IBD have an approximately 3-fold increased risk of thrombotic events, and those with active disease have abnormal blood coagulation parameters.^29^ A recent study identified an immune-coagulation gene axis in IBD in several independent cohorts where elevated PAI-1 was central in controlling key inflammatory modulators and mucosal damage in colitis.^11^ The study described that PAI-1 exacerbated mucosal damage in experimental colitis by blocking tissue plasminogen activator-mediated cleavage and activation of the anti-inflammatory TGF-β. In contrast, the inhibition of PAI-1 reduced mucosal damage and inflammation in mice. The authors also demonstrated that the colonic mucosal Serpin E1 gene expression is elevated in active, more severe IBD and patients who do not respond to anti-TNF therapy. Our results also confirmed the elevated mucosal Serpin E1 gene expression in active IBD patients. Additionally, we detected a significantly increased concentration of PAI-1 in the serum and mucosal samples of active IBD patients. Importantly, the serum and mucosal PAI-1 concentration and the Serpin E1 gene expression in the colonic tissue decreased significantly in patients who responded to anti-inflammatory therapy. Our study and the previous study by Kaiko *et al*.^11^ showed that PAI-1-positive cells were detected in the epithelium of the colonic crypts and colonic organoids, which were significantly enriched in the inflamed colon.

The costs of overall care for IBD have also increased in the past 5 years, and the annual mean health care costs are over 3-fold higher than for patients without IBD.^30^ The key cost drivers for IBD patients are treatment with specific therapeutics, including biologics, emergency department use, and health care services associated with relapsing disease, anaemia, or mental health comorbidity. Biologic, therapy-based, individualised treatment that focuses more on preventing disease progression is expensive at the outset but may ultimately lead to decreased rates of surgeries and hospitalisations, potentially yielding lower long-term costs for treatment.^31^ To achieve these goals, reliable biomarkers that are easy and relatively cheap to measure are crucially needed. Serological laboratory parameters, total leukocyte count, CRP, and erythrocyte sedimentation rate offer indirect, objective, but non-specific markers for IBD.^32,33^ In this study, both serum PAI-1 and CRP were elevated in active IBD patients. Additionally, serum PAI-1 concentration decreased significantly in therapy responder patients, whereas CRP remained elevated. Notably, a large number of studies have demonstrated relatively poor sensitivity and specificity of serum biomarkers in IBD diagnosis and patient monitoring.^32,34,35^ For example, studies showed that CRP is in the normal range in ~50% of UC patients.^35,36^ This was also confirmed in our cohort, as the patients with UC displayed significantly lower CRP levels than those with CD. Additionally, CRP can be elevated due to many factors unrelated to IBD, including infection and rheumatoid and autoimmune diseases.^35^

Recent studies indicated that faecal biomarkers strongly correlate with mucosal inflammation in IBD.^36,37^ Among many potential faecal biomarkers, most studies have been performed with FC, which is today the gold standard among faecal markers.^10^ FC is a small, calcium-binding protein found in abundance in neutrophil granulocytes.^9^ The presence of calprotectin in faeces is a consequence of neutrophil migration into the gastrointestinal tissue due to an inflammatory process; therefore, FC concentrations demonstrate a good correlation with intestinal inflammation. Several recent studies reported that the AUC of FC is between 0.8 and 0.9 in different patient populations,^38–40^ which increased above 0.9 when FC was combined with other markers, such as oncostatin M.^40^ In our experiments, faecal PAI-1 concentration was significantly higher in active IBD patients compared with the controls [AUC = 0.83] and in active versus inactive IBD and control samples [AUC = 0.82]. These results suggest that faecal PAI-1 is comparable to FC.

However, there are considerable limitations of FC in clinical settings. First, several studies demonstrated the lack of specificity of FC for IBD, and it is increased in colorectal cancer, gastroenteritis, irritable bowel syndrome, diverticulitis, food intolerance, and non-steroidal enteropathy as well.^21^ In our patient cohort, faecal PAI-1 demonstrated remarkable selectivity as it was elevated only in active IBD patients. In contrast in other GI diseases, such as adenoma, colorectal cancer, or diverticulosis, it was not significantly higher than in the control patients. In addition, FC concentration is affected by disease-independent factors such as age and comorbidities and shows a considerable day-to-day variability.^41^ There are no optimal cut-off values to define active and inactive disease or to predict clinical and endoscopic remission or treatment response.^9,42^ FC level is localisation dependent as it frequently shows normal values in CD located in the small bowel and cannot differentiate between CD and UC.^10,43^ Our preliminary results showed that faecal PAI-1 was higher in patients with colonic and ileocolonic disease localisation. Additionally it was higher in patients with ileal localisation, but the difference was not statistically significant. Therefore, this parameter requires further investigation. A recent study highlighted that FC is unstable at room temperature, which may lead to false-negative results and under-treatment in children with IBD.^44^ In contrast, faecal PAI-1 showed remarkable stability for up to 1 week.

There are some limitations to this study. First, the sample size did not allow subgroup analysis by disease type, location, and treatment type. To confirm the value of faecal PAI-1 in predicting relapses, longitudinal sample collection will be needed, which is currently ongoing. To further validate the results, multicentric clinical trials with large patient populations will be required. On the other hand, there are several strengths of the current study. First, this is the first comprehensive evaluation of PAI-1 in different human biological samples [serum, colon mucosa, and faeces] for gene and protein expression levels. In contrast, the previous report focused solely on colonic mucosal gene expression. Another strength is the identification of a potential selective faecal biomarker, which could be used in patient follow-up in IBD.

In conclusion, correct monitoring of IBD, which is necessary to guide treatment decisions in the treat-to-target era properly, can be difficult in daily clinical practice, and there is still an unmet need for biomarkers that could assess the duration of disease remission and the risk of relapse.^45^ Our results suggest that PAI-1 could be a potential novel biomarker in IBD based on the obtained data. In addition, determination of PAI-1 in the serum and faeces can overcome several limitations of the currently used biomarkers such as CRP and FC.

## Supplementary Material

jjad160_suppl_Supplementary_Tables_1-4_Figures_1-6
