# Supplementary material for: Plasminogen Activator Inhibitor 1 Is a Novel Faecal Biomarker for Monitoring Disease Activity and Therapeutic Response in Inflammatory Bowel Diseases
Source: J Crohns Colitis. 2023 Sep 26;18(3):392–405. doi: 10.1093/ecco-jcc/jjad160 (PMC10906952; doi:10.1093/ecco-jcc/jjad160)
Supplement: jjad160_suppl_Supplementary_Tables_1-4_Figures_1-6 [file jjad160_suppl_supplementary_tables_1-4_figures_1-6.docx]

**Supplementary Table 1.** Component of the completed HBSS for the generation of human colon organoids.

**Supplementary Table 2.** Substances of the human colon organoids feeding media.

**Supplementary Table 3.** The sequence, PCR product and melting temperature of the primers for the qRT-PCR analysis.

**Supplementary Table 4.** The applied softwares in this study.


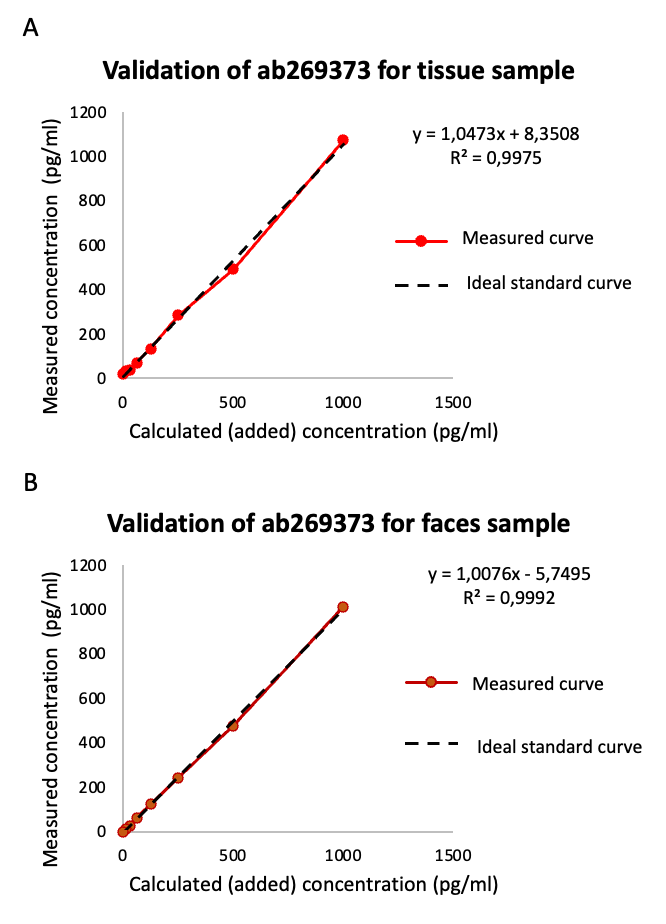


**Supplementary Figure 1.** The validation of the ab269373 ELISA kit. Segmented line shows the ideal line of the halved protein quantity, and the red line is the difference from the ideal line in percent (%). [A] Validation of tissue lysate. [B] Validation of faces samples.

**Supplementary Figure 2. Cytokine profiles in the serum and mucosa.** **A-B.** Membrane images of serum cytokine pattern in control (N=4) and IBD patients (N=9) and the summarized results. The number of independent detections of each cytokine is plotted in the different groups. The empty bars on top of the detections represent the size of the investigated samples. **C-D.** Membrane images and number of detections of the mucosal cytokines in control (N=4), non-inflamed inactive (N=15) and active inflamed (N=17) IBD patients. The empty bars on top of the detections represent the size of the investigated samples.


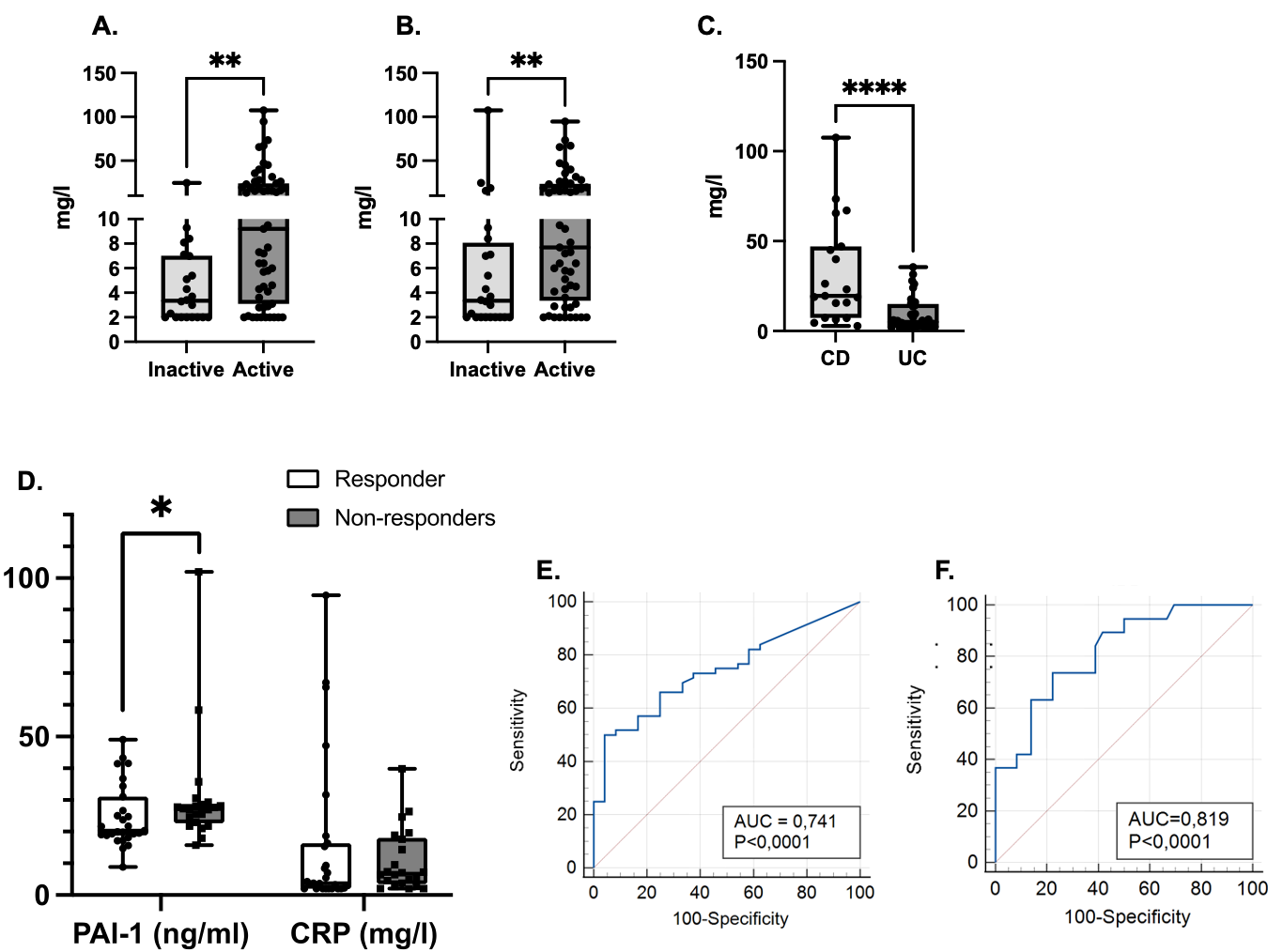


**Supplementary Figure 3. Definition of serum CRP level in IBD patients.** The CRP level was elevated in the endoscopically (**A**) and clinically (**B**) active IBD patients (endoscopically: N=55, clinically: N=53) compare to the inactive patients (endoscopically: N=22, clinically: N=24) (endoscopically inactive VS active p<0.0014, clinically inactive VS active p=0.0089). **C.** CRP was significantly higher in CD patients vs UC. **D.** The serum PAI-1 was significantly lower in responders after the treatment, whereas in the serum CRP level no significant difference was observed between the non-responders (N=27) and responders (N=22) (p=0.3634). **p<0.01. **E-F.** ROC analysis of CRP between the inactive and active IBD (E.) and CD vs UC patients (F.).


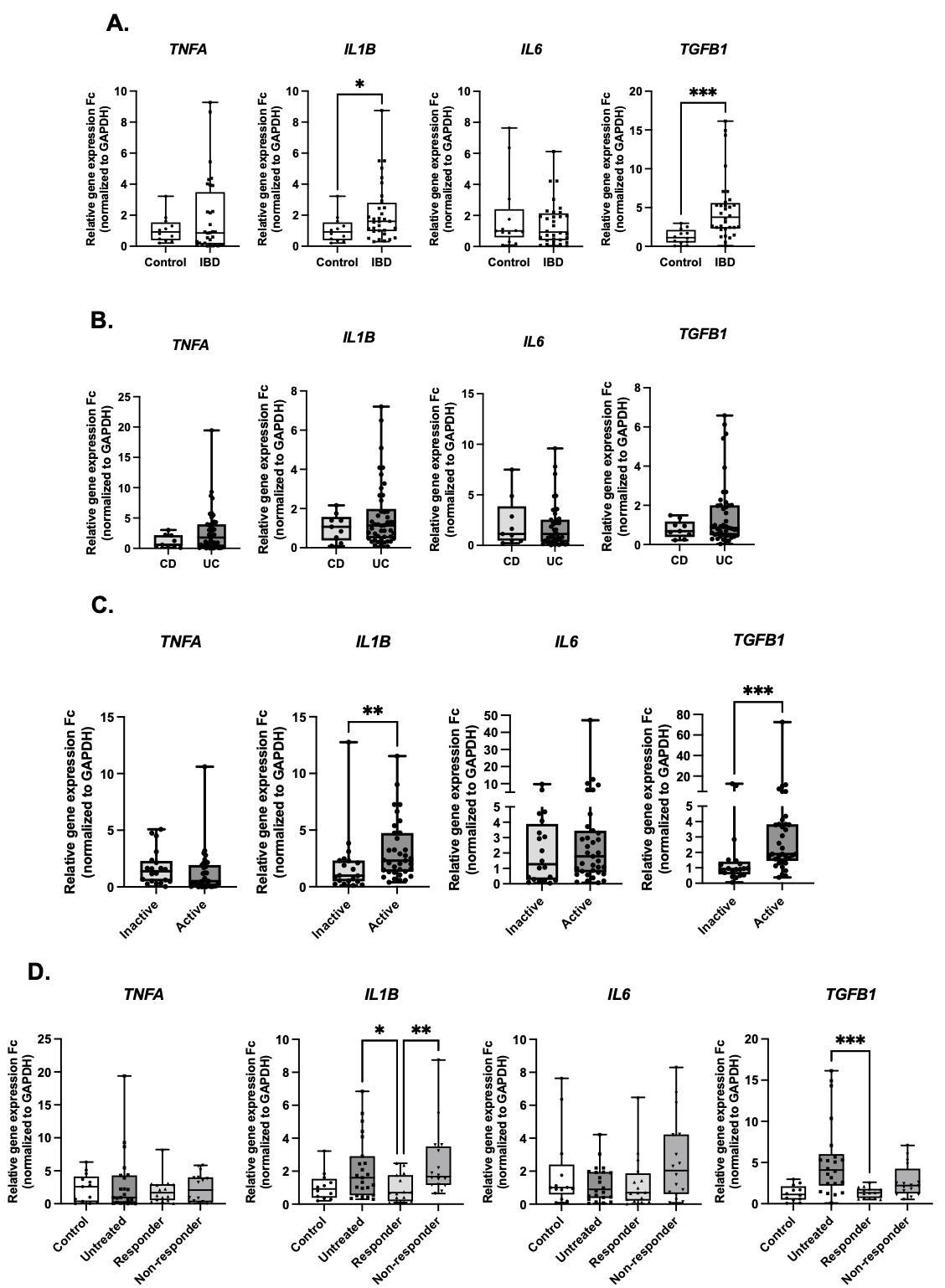


**Supplementary Figure 4. Gene expression change of pro-inflammatory cytokines in mucosa. A.** Relative gene expression fold change (Fc) in the IBD patients (N=32) compared to controls (N=13) of the TNF-α (*TNFA*), IL-1β (*IL1B*), IL-6 (*IL6*) and TGF-β (*TGFB1*). **B.** Comparison of the gene expression patterns in CD (N=12) and UC patients (N=46). **C.** Comparison of the gene expression patterns of Serpin E1 in inactive (N=20) and endoscopically active IBD patients (N=35). **D.** Comparison of the gene expression patterns in controls (N=13), untreated IBD patients (N=23), responders (N=16) and non-responders (N=16). *p<0.05, **p<0.01, ***p<0.001


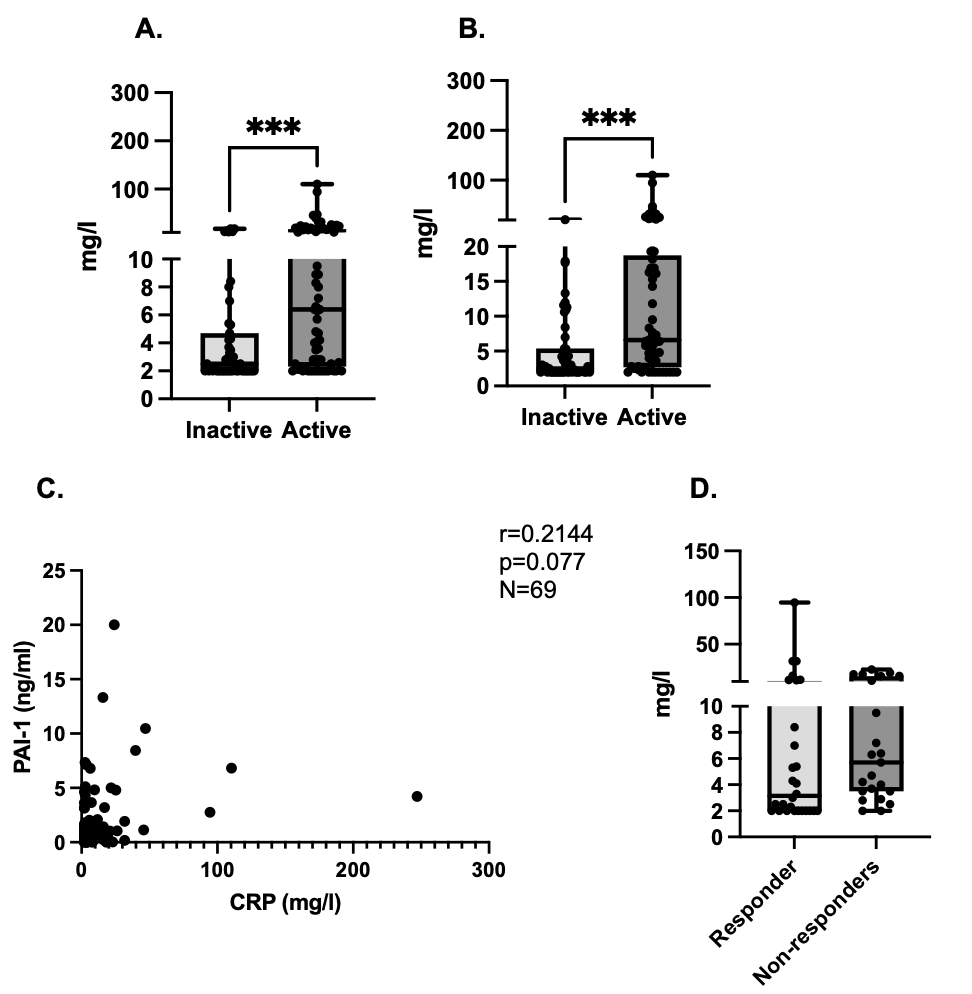


**Supplementary Figure 5. Definition of serum CRP level and comparison with faecal PAI-1 concentration in IBD patients.** The CRP level was upregulated in the endoscopically (**A**) and clinically (**B**) active IBD patients (endoscopically: N=67, clinically: N=53) compared to the inactive patients (endoscopically: N=47, clinically: N=48) (endoscopically inactive VS active p<0.0002, clinically inactive VS active p=0.0002). **C.** Correlation analysis between the serum CRP (N=69) and faecal PAI-1 level (N=69). **D.** Significant difference was not found between the non-responders (N=28) and responders (N=23) (p=0.1026). ***p<0.001


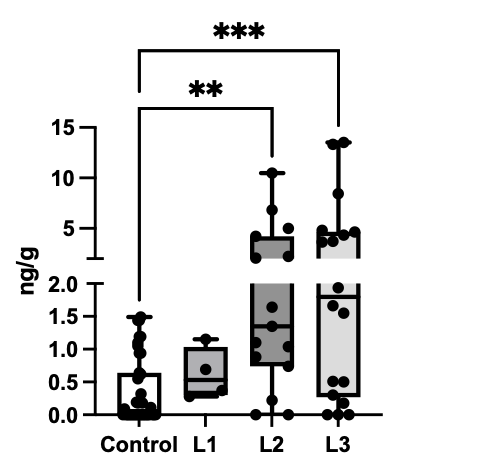


**Supplementary Figure 6. Comparison of the faecal PAI-1 level by the localization in endoscopically active CD patients** Control (N=39), L1=ileal (N=4), L2=colonic (N=15) and L=ileocolon (N=18) **p<0.01, ***p<0.001
